# Editorial: The discovery of bioactive natural products: the isolation and structural elucidation, genome mining, OSMAC strategy, and biotransformation

**DOI:** 10.3389/fmicb.2023.1253850

**Published:** 2023-08-03

**Authors:** Dewu Zhang, Kalindi Morgan, Ling Liu

**Affiliations:** ^1^Institute of Medicinal Biotechnology, Chinese Academy of Medical Sciences and Peking Union Medical College, Beijing, China; ^2^Department of Chemistry and Biochemistry, University of Northern British Columbia, Prince George, BC, Canada; ^3^Institute of Microbiology, Chinese Academy of Sciences, Beijing, China

**Keywords:** natural products, structure elucidation, genome mining, OSMAC strategy, bioactivity

Natural products play a highly significant role in the drug discovery and development process, which are massively important in medical and clinical applications. From 1981 to 2020, a total of 1,394 small molecule drugs have been approved, among them 441 (32%) were direct or derived from natural products (Newman and Cragg, 2020). Microorganisms have provided abundant sources of natural products which have been developed as commercial products for human medicine, animal health, and plant crop protection. Back in 1928, Alexander Fleming discovered penicillin from fungus *Peniciliium notatum*, which began the microbial drug era (Tan and Tatsumura, 2015). Subsequently, an increasing number of microbial drugs have been discovered, such as lovastatin, vancomycin, erythromycin, polymyxin, abamectin, and etc. Nowadays, microbial drugs have become an indispensable part of protecting human health. However, ever-increasing risks of human, animal, and plant disease and growing drug resistance require more bioactive natural products to be discovered, in order to produce relevant therapeutic drugs.

In fact, traditional isolation and structure elucidation of natural products from microorganisms are still an important approach to discover drug leading compounds, which led to the discovery of a huge amount of bioactive natural products. Recently, with the rapid development of tools for whole genome sequencing and bioinformatics analyses, it further broadens our horizons for the discovery of new bioactive compounds (Hautbergue et al., 2018).

The overall goal of this Research Topic is to propose classical and advanced strategies for the discovery of bioactive natural products, including genome-mining, one strain many compounds (OSMAC) strategy, and etc. A total of thirteen papers by 121 authors were published to represent the latest research progress in their field, which helps the field in its' understanding of these most intriguing topics.

Liu et al. reported the discovery of novel antidiabetic compounds from the fungus *Penicillium* sp. HFF16. The researchers expanded the prenylated indole-terpenoids encindolene family with the elucidation of encindolenes I–L. The isolated compounds showed inhibitory effects on glucagon-induced hepatic glucose output and decreased intracellular cAMP levels in primary hepatocytes. These findings were significant for the development of new drugs against diabetes as they provided potential candidates for suppressing the hepatic glucagon response. Song et al. isolated the twenty secondary metabolites including seven new compounds from endophytic fungus *Fusarium decemcellulare* F25. Some of them exhibited antifungal activities against plant pathogen *Colletotrichum musae* ACCC 31244. Schneider et al. investigated the structures and biological activities of suomilides, a class of highly modified non-ribosomal peptides produced by cyanobacteria *Nostoc* sp. KVJ20. The researchers isolated and determined the chemical structures of four new suomilides, studied secondary metabolite expression patterns under various growth conditions, and proposed a biosynthetic gene cluster for the production of the suomilides. The study contributed to the understanding of the biosynthesis of suomilides and expanded the knowledge of cyanobacterial secondary metabolites. Wu et al. presented the isolations and structure elucidations of five new dimeric tetrahydroxanthone derivatives and four known dimeric tetrahydroxanthone analogs from the marine-derived fungus *Aspergillus aculeatinus* WHUF0198, and some of them displayed cytotoxic and antibacterial activities.

Yu et al. reported the discovery and biosynthesis of macrophasetins from the plant pathogen fungus *Macrophomina phaseolina*. The authors mined a PKS-NRPS and LLDAse encoding gene cluster from fungus *M. phaseolina* using bioinformatics analysis prediction and heterologous expression, and characterized the cluster to be responsible for the biosynthesis of novel 3-decalinoyltetramic acids (DTAs), macrophasetins. The DTA biosynthesis pathway were also investigated, and the results further validated the accuracy of the bioinformatics prediction. The findings set a good example to mine novel natural products from fungi by the combination of bioinformatics prediction and heterologous expression. Wang Z.-J. et al. exploited an efficient heterologous system based on streamlined *Burkholderia thailandensis* E264. The disorazol and rhizoxin biosynthetic gene clusters were expressed successfully with this host, and the yield of the heterologous product, disorazol F_2_, was improved 96-fold by promoter substitution and insertion. This heterologous expression system provided another choice for the exploration of natural products from different phylogenetic taxa.

Qu et al. investigated the bioactivities and metabolomics of *Cordyceps gunnii* under different culture conditions. Five different solid media were used to culture the *C. gunnii* mycelium, and the extracts were assayed for various biological activities and analyzed using untargeted metabolomics. The results showed significant differences in activities and metabolites among the extracts from different culture conditions. This study provided a basis for further OSMAC applications to macrofungal species, contributing to the field of macrofungal secondary metabolite research. Wang Y. et al. reported the metabolomic profiles of the fermentation in co-culture of *Eurotium amstelodami* and *Bacillus licheniformis*. The results revealed that the co-culture combination of *E. amstelodami* and *Bacillus* species could significantly improve antibacterial activity against *Staphylococcus aureus*. Metabolomics data further indicated that abundant and various secondary metabolites were significantly enhanced in co-culture. This is the first metabolomics-based report of metabolite profiles of *E. amstelodami*. This study provided novel insights into the selection of good co-culture combinations, as well as activation of silent microbial secondary metabolite biosynthetic gene clusters. Lu et al. found a marine fungus *Penicillium mallochii* ACD-5 from sea cucumber with high potential in alkaloids production by *in situ* colony assay, TLC chemical colorization, and LC-MS/MS based metabolomics profiling. Using comprehensive metabolic annotation strategy, three chlorinated azaphilone alkaloids were obtained from *P. mallochii* ACD-5, and one of them showed remarkable anti-neuroinflammatory activity in liposaccharide induced BV-2 cells.

Duan et al. investigated the genome, transcriptome, and metabolome analysis of edible fungus *Dictyophora indusiata* (DI). Authors firstly created DI reference genome and identified 19,909 coding genes. Subsequent transcriptome and metabolome analyses of the five tissues of the DI fruiting bodies and mycelium indicated the mechanism underlying DI fruiting body differentiation, and also identified 728 metabolites. Meanwhile, the authors confirmed the significance of tryptophan metabolism and metabolite synthesis related genes in regulating fruiting body formation. This study expanded the understanding of resource development and application for DI.

Zhang et al. studied the molecular mechanism GylR-mediated regulation of glycerol metabolism in *Streptomyces clavuligerus* NRRL 3585. Authors identified the effector molecules and binding sequences of GylR, and further identified a minimal essential operator site (a core 18-bp sequence motif) of GylR-like regulators. The findings provided new basic elements for the development and application of glycerol-inducible regulatory tools for microbial metabolic engineering and synthetic biology research.

Morgan reviewed the use of nitrogen-15 in bacterial and fungal natural product discovery and biosynthetic characterization. Nitrogen-15 is an important element in various bioactive and structurally intriguing natural products, including alkaloids, non-ribosomal peptides, and hybrid natural products. The article highlighted the detection of nitrogen-15 at natural abundance using nuclear magnetic resonance and mass spectrometry techniques. It also explored the use of nitrogen-15 stable isotope feeding for biosynthetic characterization. The article provided insights into the role of nitrogen-15 in natural product discovery and characterization. Ren et al. reviewed the discovery and excavation of lichen bioactive natural products. The authors summarized the lichen-derived bioactive metabolites and highlighted the application of OSMAC, molecular network, and genome mining-based strategies in lichen-forming fungi, which offer great help to discover numerous novel secondary metabolites with various biological activities.

Overall, the works presented in this Research Topic provide an overview of some of traditional and latest strategies for the discovery of bioactive natural products. In order to cope with the growing risk of disease, it is necessary to further explore bioactive components with multiple pharmacological activities. However, the research and development of most of natural products is only in preliminary *in vitro* experimental stage. So, the more in-depth pharmacological experiments for natural products need to be carried out for natural product research. We sincerely hope that this Research Topic can increase awareness and serve as forward-looking guide in the field of microbial natural products.

## Author contributions

DZ: Writing—original draft, Writing—review and editing, Funding acquisition. KM: Writing—original draft, Writing—review and editing. LL: Funding acquisition, Writing—original draft, Writing—review and editing.
